# Effects of Using Guided Deep Breathing Exercises in a Virtual Natural Environment to Reduce Stress during Pediatric Treatment

**DOI:** 10.3390/healthcare11243140

**Published:** 2023-12-11

**Authors:** Ilmari Jyskä, Markku Turunen, Arash Chaychi Maleki, Elina Karppa, Sauli Palmu, Jari Viik, John Mäkelä, Kaija Puura

**Affiliations:** 1 TAUCHI Research Center, Faculty of Information Technology and Communication Sciences, Tampere University, FI-33014 Tampere, Finland; markku.turunen@tuni.fi (M.T.); arash.chaychi@gmail.com (A.C.M.); john.makela@tuni.fi (J.M.); 2 TamCAM Research Center, Faculty of Medicine and Health Technology, Tampere University, FI-33014 Tampere, Finland; elina.karppa@tuni.fi (E.K.); sauli.palmu@tuni.fi (S.P.); kaija.puura@tuni.fi (K.P.); 3 Department of Pediatrics, Tampere University Hospital, Central Hospital, P.O. Box 2000, FI-33521 Tampere, Finland; 4 Finnish Cardiovascular Research Center, Faculty of Medicine and Health Technology, Tampere University, FI-33014 Tampere, Finland; jari.viik@tuni.fi; 5 Department of Child Psychiatry, Tampere University Hospital, Central Hospital, P.O. Box 2000, FI-33521 Tampere, Finland

**Keywords:** virtual reality, child, anxiety, stress, nature, analgesia, virtual natural environments, deep breathing, heart rate variability

## Abstract

There exists a need for new methods to address treatment anxiety in pediatrics—at the same time, deep breathing exercises and virtual natural environments have both been known to have stress-reducing qualities. This article reports the combined effect of these two methods in a pediatric setting. A feasibility study was conducted in a local hospital. The study had a within-subjects design, and it included 21 child patients aged 8 to 12 years old, who used a virtual reality (VR) relaxation application developed for this purpose during an intravenous cannulation procedure related to their treatment. The key findings highlight a statistically very significant stress reduction associated with the utilized VR intervention, demonstrated by heart rate variability measurements (SDNN, *p* < 0.001; RMSSD, *p* = 0.002; Stress Index, *p* < 0.001; LF/HF ratio, *p* = 0.010). This effect was consistent regardless of the level of general anxiety or the level of needle phobia of the patient, and no adverse effects were observed. The results show the strong potential of using deep breathing exercises in virtual natural environments for addressing treatment anxiety related to invasive pediatric procedures.

## 1. Introduction

The issue of stress, manifested as pain and anxiety, is a notable concern in pediatric treatment. This can result in severe physical reactions during invasive procedures and significant anxiety about future medical interventions. Consequently, this may contribute to a reluctance to seek treatment in adulthood [1,2]. Recent research [3,4,5,6] indicates that modern virtual reality (VR) solutions hold promising potential for addressing this issue, although there is a limited number of studies involving child patients. While studies generally endorse the use of VR methods in pediatrics for reducing pain and anxiety, an ongoing scientific debate surrounds the choice of VR content in this context. The discussion primarily centers on the concept of distraction by VR and whether the content should be passive or interactive. However, this perspective oversimplifies the nature of VR content—VR applications can incorporate both passive and interactive elements simultaneously. Additionally, when using a head-mounted display (HMD), even seemingly passive VR content typically involves a system output that resembles interactivity through the visual view being controlled by the user’s head movements.

This article explores the effectiveness of an innovative VR relaxation approach, which integrates both passive and interactive elements to alleviate stress and anxiety levels in 8-to-12-year-old child patients undergoing an intravenous cannulation. The VR relaxation method centers on established anxiety reduction techniques—specifically deep breathing [7] and immersing the child in a calming natural environment [8]. The emphasis lies on proven anxiety reduction methods rather than solely relying on VR distractions or the extent of the interaction with the VR content.

A feasibility study was conducted at a local hospital involving 21 child patients undergoing cannulation while utilizing an HMD to engage in a deep breathing exercise within a virtual natural setting. Positive findings were achieved in the study, addressing both anxiety reduction and user experience. The focus of this article is on examining the stress-reducing effects of this VR relaxation method.

However, another article by the authors [9] showcases the design process in detail along with a user-experience study. The key findings of that article included positive user experiences among both the child patients and pediatricians. Furthermore, while most patients found the method useful, participants with increased levels of general anxiety or needle phobia reported the highest scores in usefulness. This indicates a good potential to address treatment anxiety with this method. The findings also recommend a multidisciplinary design approach when developing VR applications for pediatrics. In practice, this means VR developers working directly with pediatricians during the design process. The results also suggest that the level of interaction with the VR application should not be used as a guideline when selecting VR methods for stress reduction in pediatrics.

## 2. Background

The fear of an upcoming procedure or needles has been treated by distraction or educating the patient about the upcoming situation [10]. The treatment may include sedative medication—most commonly Midazolam, propofol, or dexmedetomidine—but the use of sedative medications places the patient at risk of adverse effects [11].

Utilizing VR methods for anxiety reduction in healthcare services provides a non-pharmacological and non-invasive alternative to more traditional methods, as highlighted in the studies [12,13,14]. Furthermore, VR methods have proven to be successful in mitigating pain and distress associated with treatment [15,16] while being generally well-received by both child patients [17] and healthcare professionals [18]. Additional advantages include cost-effectiveness and economy of scale [19,20], repeatability, and a minimal risk of adverse effects [21]. However, the challenges include the necessity for a true multidisciplinary collaboration in therapeutic VR application developments [22] and the relatively low technical proficiency among medical staff necessitating training and technical support for VR equipment use [18,23]. Additional considerations involve the possibility of cybersickness-induced nausea [24,25] and the expected gradual decrease in the novelty value upon repeated use [23]. Moreover, VR technology allows for the digital replication of established stress-reduction methods in a treatment setting where these approaches might otherwise be too resource-demanding, impractical, or entirely unfeasible in pediatric care. Replicating the positive effects of being in a serene forest environment [26] is a good example.

Indeed, the recent research indicates that employing virtual natural environments—digitally replicated natural settings—is likely to have a positive impact on stress and anxiety levels, akin to the effects observed in real natural environments [27,28,29,30]. While most related studies appear to concentrate on virtual forest environments, similar positive effects have been observed in studies exploring other virtual natural environments. For instance, environments featuring water landscapes also demonstrate efficacy in inducing stress relief. [31,32]. In addition, the positive findings regarding virtual desert environments by Yin et al. [33] indicate that past experiences and familiarity contribute to the health benefits associated with these virtual natural environments. One of the advantages of virtual natural environments is the potential to integrate them with other relaxation methods.

Deep breathing is a common, effective, and highly cost-efficient method for promptly reducing stress and anxiety levels, while also promoting emotional self-regulation [7,34]. However, it is not widely employed in pediatric care, where medication is often used for similar purposes [35]. The limited adoption of deep breathing in pediatric care may be attributed, in part, to the practical challenges and concerns about treatment consistency, such as the absence of validated procedures for deep breathing in this context. Nevertheless, a growing body of scientific evidence supports the efficacy of deep breathing exercises in virtual natural environments to effectively alleviate stress and anxiety levels [36,37,38]; however, the research involving patients in pediatric care remains notably limited.

An established method to measure such effects is heart rate variability (HRV). HRV measurements have gained popularity as an objective research method for evaluating the possible changes in a subject’s stress and anxiety levels. HRV is a measure of the variation in time between each heartbeat, and it can be used to gain insights into how the autonomic nervous system (ANS) of the subject is operating in the studied timeframe, which in turn can be used to objectively evaluate the subject’s state of mind, especially regarding the changes in the state of relaxation or stress experienced [39,40].

Building on this scientific foundation, the authors developed a therapeutic VR application centered around virtual natural environments and deep breathing guidance. The objective was to investigate whether this method could serve as a novel tool for pediatricians to diminish anxiety and fear associated with treatment in child patients.

## 3. Materials and Methods

A feasibility study of this method was conducted in a local hospital with 21 Finnish child patients. The study used a relaxation application (VirNE) designed for this research, and the research protocol was adapted from a previous user-experience study involving HRV data and an HMD-meditated virtual reality relaxation application [41].

The protocol was based on evaluating both subjective and objective data regarding the effect of the method studied, both from the human–computer interaction and medical points of view. The subjective data collected in this study included questionnaires, interviews, and observations from nurses conducting the research, while the HRV data, which could be regarded as objective data, were collected to evaluate possible changes in the stress and relaxation levels of the participants.

### 3.1. Virtual Natural Environments (VirNE) Application

The virtual natural environments (VirNE) application aims to reduce stress and anxiety levels in children aged 8–12 years old using virtual reality (VR). It combines 360-degree videos of natural environments with guided relaxation exercises. The application is used with a Meta Quest 2 HMD [42] and controlled by a computer via Oculus Link [43].

VirNE targets children experiencing stress and anxiety, and the effect of a deep breathing exercise on the patient’s stress levels is evaluated in this article. VirNE uses dialog, avatar characters, and animations to guide users through exercises while using 360-degree still videos of Finnish natural environments and a stereo sound file of gentle nature sounds to deliver the virtual natural experience.

**Figure 1:**
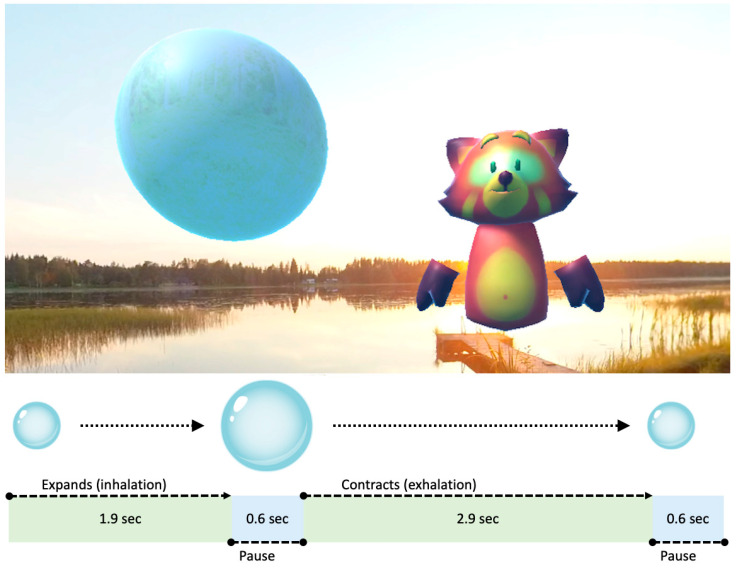
The breathing balloon animation and a guiding avatar character in a VirNE exercise, along with an explanatory breathing animation diagram.

The deep breathing exercise studied in this article lasted for six minutes and included audiovisual cues for breathing rhythm in the form of an animated breathing balloon, which expanded and contracted in the rhythm of the desired breathing cycle. According to Zaccaro et al. [34], the optimal breathing frequency for adults is six breaths per minute, which is about half of the usual breathing frequency. Using this principle, for 8 to 12 year olds, the optimal breathing frequency was calculated to be 10 breaths per minute. Figure 1 presents the patient view and the breathing balloon animation cycle used in the exercise.

The exercise also includes a pre-designated timeframe for performing the medical procedure. A more detailed structure of the deep breathing exercise used in this study can be seen in Table 1.

The application was operated through Oculus Link, and the research participants selected environments and guiding avatar characters before wearing the VR headset. Five different virtual environments were used in this research, which were based on Finnish nature and cultural context. Two of these included a clear water element, while three were based on different kinds of forest scenery. The avatar character selection included three options: two animated characters from Mozilla Hubs [44] and a possibility to select no avatar character. In the latter case, the exercise guidance dialog was audible but had no visual counterpart.

Another article by the authors [9] featured a more detailed description of the VirNE application and showcased its multidisciplinary design process.

### 3.2. Heart Rate Monitor Application

To meet the ethical requirements of medical research regarding child patients, the HRV data collected in the study needed to be stored locally, without the involvement of a 3rd party, such as a company providing the application to collect the HRV data. In addition, the research protocol needed an accurate and reliable way to mark certain events in the HRV data. For these reasons, an Android application named Heart Rate Monitor was developed for this purpose. The development was conducted in collaboration with pediatricians and human–computer interaction experts.

Heart Rate Monitor is an Android application based on Polar SDK [45], and it uses a Bluetooth connection to establish a link with a Polar H10 heart rate sensor. The HRV data collected are stored locally on the device running the application and consist of numerical data regarding RR intervals in ms units. In addition, Heart Rate Monitor creates a metadata file per each HRV recording, which includes data regarding user-selected points of interest during the recording. In practice, this meant the possibility to mark the timestamp of the following distinct moments during the HRV recording: the operational HMD device on the participant, the commencement of the deep breathing exercise, the medical procedure (cannulation), and the end of the deep breathing exercise.

### 3.3. Research Methods in Brief

**Figure 2:**
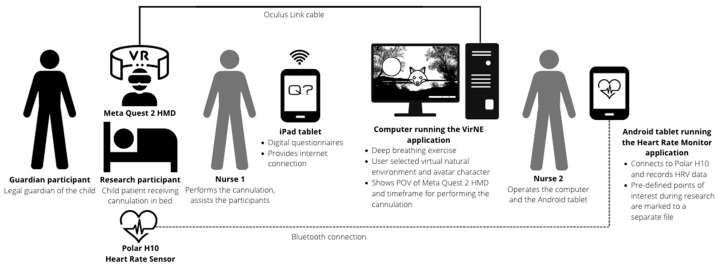
Research scenario. Child patient receiving the treatment is lying on a bed in a half-sitting posture, wearing a Polar H10 chest strap, and is using a Meta Quest 2 HMD to experience the deep breathing exercise during the cannulation procedure.

The feasibility study was conducted in the pediatric clinical trials unit of a local hospital. The study was supervised and conducted by pediatric experts and consisted of research using 21 Finnish child patients aged between 8 to 12 years old. These research participants used the VirNE deep breathing exercise in a controlled setting to reduce pain and anxiety while receiving cannulation as part of their normal treatment requiring an intravenous catheter, as presented in Figure 2. The intravenous catheter was required for administering either intravenous medication for treatment or for anesthesia for medical imaging. The guardians of the participating child patients were present in the research scenario and participated by supplying background information and filling out a questionnaire regarding the level of anxiety of their children. This study was conducted in the Finnish language and all English translations were performed by the authors.

### 3.4. Participant Enrollment and Research Preparations

Suitable participants for this research were pre-selected from the patient records of the hospital. Inclusion criteria for the research were Finnish 8-to-12-year-old literate child patients, who were to receive an elective cannulation as part of their treatment with a suitable schedule. Exclusion criteria for the research included various medical conditions, such as epilepsy, vertigo, unstable heart condition, and problems regarding their hearing ability or eyesight. Potential participants and their legal guardians were contacted in person on the day of their scheduled treatment in the hospital, introduced to the study, and offered the possibility to participate in it.

The research situation was operated by two nurses who both controlled the research equipment and performed the cannulation. Before the research participants entered the research room, the technical functionality of the equipment used was prepared and verified. This included setting up Meta Quest 2 HMD with a room-scale boundary and suitable default point of view, setting up the computer running the VirNE application and the Oculus Link connection, setting up an iPad for the Internet connection and digital questionnaires used in the research, and setting up an Android tablet running the Heart Rate Monitor application.

### 3.5. Pre-Procedure Phase of the Research

The data gathering of the research situation was divided into three phases: pre-procedure, VR intervention and cannulation, and post-procedure. In the first phase, the research started with nurses measuring the height and weight of the child participant and collecting detailed background data by interviewing the participants. These data included information about the child participant regarding his/her previous experiences of cannulations, breathing exercises, and virtual reality applications, as well as detailed information about his/her medical conditions and hobbies.

The subsequent steps included assisting the child to wear the Polar H10 chest strap and connecting it to the Heart Rate Monitor application. After the connection was established, the patient was directed to make a half-sitting posture in a hospital bed, and the HRV recording was started. The pre-procedure phase of the HRV recording was used to collect the baseline HRV data from the child participants. As with all phases in the research, this phase had a minimum length of 5 min to ensure enough HRV data were collected for future analyses. Furthermore, it should be noted that the child participant stayed in this posture until the end of the post-procedure phase.

The pre-procedure phase continued with clinical data measurements, including blood pressure, oxygen saturation, body temperature, and breathing frequency; however, these data were excluded from the results of this article. After these measurements were performed, the patient was given an iPad with digital questionnaires to fill out. These questionnaires included a 41-point SCARED [46] questionnaire for children to measure the level of anxiety of the participant and an adapted 2-point VAS-A [47] questionnaire to measure the expected level of anxiety and pain during the procedure.

Then, the iPad was given to the legal guardian of the child who proceeded to answer a SCARED questionnaire for parents, while the nurses prepared the cannulation for the child. This preparation included selecting the location for the intravenous catheter and explaining the procedure to the patient, who could select whether they wanted a tourniquet needed for the procedure to be prepared at this point or during the VR intervention phase. In both cases, the final adjustment of the tourniquet was performed during the VR intervention phase, just before the cannulation.

When everything was ready, the patient selected the desired virtual environment and avatar character for the VR intervention from a set of pictures presented by a nurse, before being assisted to wear the HMD used for the VR intervention. Then, the study proceeded to the VR intervention and cannulation phase.

### 3.6. VR Intervention and Cannulation Phase of the Research

In this phase, nurses marked four data points of interest for the HRV recording in the Heart Rate Monitor application. The first mark was entered when this phase started. In other words, a nurse marked the point when the child participant wore the HMD, before proceeding to verify that the Oculus Link connection between the HMD and the computer running the VirNE application was operational. Then, the application parameters were set up according to the patient’s selections, and the deep breathing exercise was initiated. Exercise start was the second point of interest in the HRV recording.

Before the cannulation, the nurses observed the exercise in silence, unless being talked to, and measured the respiration frequency of the child. During the timeframe marked for performing the cannulation, one nurse performed the cannulation as close to a standard medical procedure as possible, including informing the child of what was happening, while the other nurse marked the point of cannulation as the third point of interest in the HRV recording. In case the cannulation failed, the participant was given the opportunity to receive the cannulation without a VR intervention or to continue with the exercise along with a new attempt to cannulate.

When the exercise was completed, the fourth and last point of interest was marked in the HRV recording. Then, the nurses removed the HMD from the child participant and the post-procedure phase of the research started.

### 3.7. Post-Procedure Phase of the Research

This last phase started with a very brief unstructured interview about the child participant’s thoughts and feelings about the exercise and cannulation. Then, the iPad with digital questionnaires was handed to the child. The questionnaires of this phase consisted of an adapted 2-point VAS-A questionnaire to measure the experienced levels of anxiety and pain during the procedure and a user-experience questionnaire.

When the patient had completed the questionnaires, the medical data measurements conducted in the pre-procedure phase were measured again. Subsequently, the nurses verified that this post-procedure phase lasted for at least 5 min, and if the time was reached, the nurses ended the HRV recording. If not, the nurses talked informally with the child, until 5 min had passed and the HRV recording could be stored.

Then, the child participant was asked to stand up and the heart rate sensor was removed, and both the child and legal guardian could comment on and ask questions about the research. If their comments revealed information of interest regarding the research situation, it was marked in the research documents. Finally, the nurses ended the data collection phase of the research and the patient could continue with normal treatment procedures related to the intravenous catheter they received during the research.

### 3.8. Measures

This section of the article presents the details about the measures employed in the research. The information presented here focuses on the data relevant to this article, excluding the majority of medical data and information related to user experience collected during the research. However, the data regarding user experience are available in another article by the authors [9]. All questionnaires utilized in the study were digital versions in Finnish, created with Microsoft Forms. Research participants entered the data using a tablet during the research session.

#### 3.8.1. SCARED Questionnaire (Screen for Child Anxiety Related Emotional Disorders)

The SCARED questionnaire was employed to assess symptoms associated with anxiety disorders and phobias [46]. This questionnaire consisted of 41 statements addressing unpleasant emotions in various everyday situations, accompanied by a query about whether the individual experienced these emotions constantly, occasionally, or never. These statements could be directed either to the child or to the child’s adult guardian. In this study, both approaches were utilized in the pre-procedure phase. The child participant completed the SCARED for children questionnaire, while the legal guardian of the child completed the SCARED for parents questionnaire. The objective was to gather information on the potential impact of the assessed level of anxiety on other measured data in this research.

#### 3.8.2. Adapted VAS-A Questionnaires (Visual Analogue Scale for Anxiety)

The VAS-A is a validated [47,48] visual scale used to assess the intensity of pain or anxiety [49]. In this research, adapted digital versions of VAS-A regarding both pain and anxiety were used in the pre- and post-procedure phases of the research.

During the pre-procedure, the child participant was asked to rate the expected pain and anxiety during the procedure, while in the post-procedure, the questions referred to what the participant experienced during the procedure. The questions were formatted as “How much [pain/anxiety] [do you expect to experience/you experienced] during the procedure?”. Both versions included 2 questions with a 7-point Likert scale (0–6), where the answer 0 was labeled as “Not at all” and 6 as “extreme [pain/anxiety]”.

This adaption was based on previous user-experience research regarding a VR relaxation application [41]. It provided insights into how the VR intervention affected the experience in comparison to participant expectations and offered a possibility to study the possible correlations between these expectations and experiences in relation to other data collected in this research.

#### 3.8.3. Heart Rate Variability (HRV) Data

HRV is a measure of variation in time between each heartbeat, and it can be used to gain insights into how the autonomic nervous system (ANS) of the subject is operating in the studied timeframe, which in turn can be used to objectively evaluate the subject’s state of mind, especially regarding the level of relaxation or stress experienced [39,40]. This study collected HRV data from the child participant in all three phases with a Polar H10 Heart Rate Sensor, and the data were stored locally with an Android tablet running the Heart Rate Monitor application.

The HRV data variables chosen for this research were the standard deviation of normal-to-normal intervals (SDNN), root mean square of successive differences between normal heartbeats (RMSSD), Stress Index (SI), and the ratio of low frequency to high frequency (LF/HF ratio). The values of SDNN and RMSSD are related to the activity of the parasympathetic nervous system and provide information regarding the level of relaxation of the subject [50]. Both values correlate positively with the level of relaxation, with higher values indicating a higher state of relaxation. The Stress Index (SI) is related to cardiovascular system stress and the sympathetic nervous system [51]. High SI values imply reduced HRV and a stressful experience, while low SI values suggest a more peaceful experience. Finally, although being criticized for inaccuracies and ambiguity, the LF/HF ratio has been widely used to measure shifts from the sympathetic dominance to parasympathetic dominance of ANS and vice versa, where increases in the value refer to increased stress and decreases indicate increased relaxation [52,53].

**Figure 3:**
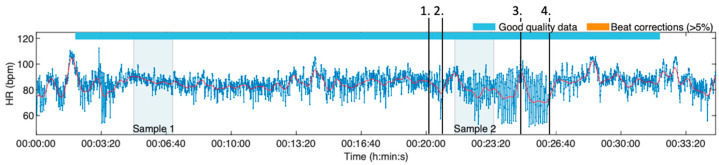
This HRV data example demonstrates the two samples analyzed in this research article. Four points of interest are marked on the graph: 1. HMD device equipped, 2. Start of the deep breathing exercise, 3. The procedure (cannulation), 4. End of the exercise.

In this article, two points of interest were studied. As Figure 3 illustrates, the first sample was obtained in the pre-procedure phase, and it consisted of HRV data between five and seven minutes into the HRV recording. This sample was considered as a baseline value. The second sample consisted of a timeframe starting at 30 s into the VirNE deep breathing exercise. Both samples were two minutes long and both occurred before the actual cannulation procedure. The aim was to analyze the possible effects of the virtual reality exercise before the cannulation procedure affected the data.

#### 3.8.4. Measures from Interviews

The following measures were derived for this article from the participant interview data: the level of needle phobia [54] and general user experience. These measures were evaluations from the interview data conducted by the main authors of this article.

In this study, the level of needle phobia was measured with a Likert scale (0–2). Value 0 equaled no needle phobia, whereas value 1 referred to minor signs of needle phobia, and value 2 to clear signs of needle phobia. These data were obtained from the pre-procedure interviews with participants and their legal guardians and were used to evaluate the possible effect of needle phobia on the other results.

Furthermore, general user experience is a measure with three possible values for the experience per participant: positive, neutral, and negative. These data were evaluated from the post-procedure interviews and were used to support the analysis of other variables in the study.

### 3.9. Research Ethics Approval

This study was approved by the Authority Ethics Committee of Pirkanmaa Wellfare Area (R21068L) and Finnish Medicines Agency (2021/007366).

## 4. Results

This research was conducted with 21 child patients and their legal guardians. However, two participants were identified as clear outliers during the evaluation and were therefore excluded from the statistical results. Among these two instances, one involved a participant who fainted in the post-procedure phase and had a history of fainting during the same medical process. The other case involved a participant who struggled or chose not to focus on the VR content, persistently attempting to observe the actual surroundings through the narrow gap between the face and the bottom of the HMD. The demographics of the 19 child participants included in the results are presented in Table 2.

The initial attempt to cannulate was unsuccessful for three participants. Subsequently, these participants were given the option to either continue with the VR intervention or remove the headset before the second attempt. All three participants expressed a desire to continue with the VR intervention and were included in the results. No adverse effects caused by the VR intervention were observed in the study.

### 4.1. Questionnaires

This section presents the results from the questionnaires used in this article. All questionnaire results include data from 19 participants.

#### 4.1.1. SCARED Questionnaire (Screen for Child Anxiety Related Emotional Disorders)

**Figure 4:**
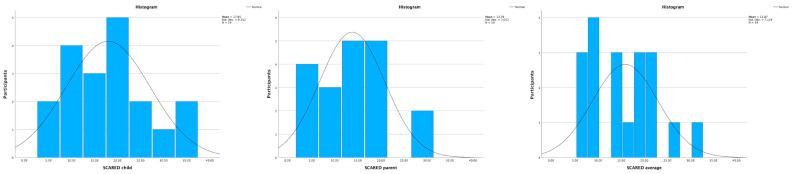
SCARED questionnaire results with histograms and a line presenting the normal distribution. All three graphs indicate a non-normal distribution of the data.

The median total score (IQR) for the SCARED questionnaire for children was 18.0 (14.0), while for the SCARED questionnaire for parents, it was 13.0 (10.0). A total score of 25 or higher may indicate the presence of an anxiety disorder [46]. According to the answers from the children, four of them were above this threshold, while only two sets of answers from the legal guardians presented a value higher than 25. As the results slightly differed between these two questionnaires, the average values obtained from these two questionnaires were estimated to be the most reliable way to analyze the results. The median value (IQR) for the SCARED average was 15.0 (12.0). Further details can be seen in Figure 4.

A Spearman’s rank-order correlation was run to determine the relationship between the data from SCARED child and parent questionnaires. There was a moderate positive correlation between the answers, which was statistically significant (*r_s_*(17) = 0.480, *p* = 0.037). In other words, the SCARED answers from legal guardians tended to have lower scores than answers from child patients, but the results were otherwise similar. This supported the decision to use SCARED average data for the analysis, but also suggested that legal guardians might have underestimated the anxiety levels of their children in general.

The participants in this study could be evaluated to be diverse in terms of level of anxiety but somewhat skewed towards low anxiety, and had relatively good mental health in general. The SCARED average data were compared to other variables in the Correlations Section in this chapter in order to evaluate whether the level of anxiety measured by SCARED questionnaires affected the other variables.

#### 4.1.2. Adapted VAS-A Questionnaires (Visual Analogue Scale for Anxiety)

**Figure 5:**
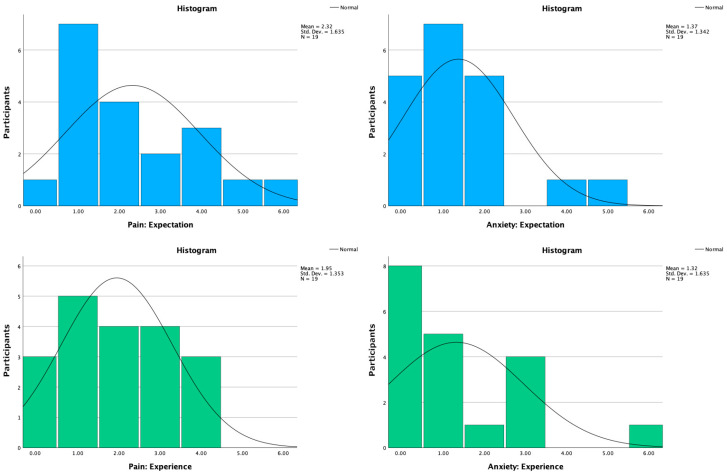
Adapted VAS-A results. These histogram charts represent the sum of participants per value regarding the question. Value 0 represents “not at all” on a 7-point Likert scale, while value 6 represents “extreme pain/anxiety”.

The VAS-A questionnaire answered in the pre-procedure phase of the research had median scores (IQRs) of 2.00 (3.00) regarding expected pain caused by the cannulation and 1.00 (2.00) regarding expected anxiety experienced during the procedure. These values indicate a relatively low expectation regarding pain and low anxiety expectations. The post-procedure answers had median scores (IQRs) of 2.00 (2.00) regarding pain experienced and 1.00 (3.00) regarding anxiety experienced. Figure 5 illustrates these results and the scale used for the measurements, along with question-specific histograms.

When comparing the mean values of these conditions from expectations to experiences, a 15.9% decrease in pain and a 3.6% decrease in anxiety were observed. Wilcoxon signed-rank tests found no statistically significant differences between the conditions regarding pain (*z* = −0.818, *p* = 0.414) or anxiety (*z* = −0.663, *p* = 0.507). In this article, future references regarding the changes in the VAS-A questionnaire results from expectations to experiences were labeled as pain change and anxiety change, with negative values indicating a positive effect.

A closer inspection of the results reveals a visible shift towards less pain experienced than expected, as can be observed from the histograms. The highest value for experienced pain was 4 out of 6, so while cannulation is a painful medical procedure, none of the participants seemed to have experienced major pain. However, regarding anxiety, the results are more mixed. While the majority (13) of the participants reported experiencing only little or no anxiety, reducing the mean value to negative in comparison to expectations, four participants reported medium anxiety and one reported extreme anxiety—which as an increase from their expectations.

### 4.2. Heart Rate Variability (HRV) Measurements

The HRV data included measurements from 18 participants. The data from one participant was excluded due to corrupted HRV data. The analysis of the HRV data was based on the study of four measures related to HRV: SDNN, RMSSD, Stress Index (SI), and LF/HF ratio. Two two-minute samples were compared in the analysis, including a baseline sample and a VR intervention sample. The median values and interquartile ranges (IQRs) of these variables regarding the two samples studied are presented in Table 3, which also shows the HR (heart rate, beats per minute) as the first variable.

The Wilcoxon signed-rank tests achieved a statistically very significant difference between baseline and VR intervention conditions regarding all four key HRV measures: SDNN (*z* = −3.724, *p* < 0.001), RMSSD (*z* = −3.157, *p* = 0.002), Stress Index (*z* = −3.724, *p* < 0.001), and LF/HF ratio (*z* = −2.591, *p* = 0.010). Regarding heart rate, no statistically significant difference was observed between the conditions (*z* = −1.635, *p* = 0.102).

In the VR intervention, the SDNN value was higher and the stress index lower than in the baseline measurements for all 18 participants, which was a clear indication of reduced stress and increased parasympathetic activity. However, regarding the RMSSD and LF/HF ratio values, the results were more mixed. While 14 participants presented “positive” results for these values (increased RMSSD and decreased LF/HF ratio values), a total of four participants presented a “negative” result for either the RMSSD or LF/HF ratio and two of them presented a “negative” result for both variables.

These four participants also presented at least some indication of negative feelings towards the experience in the subjective data collected in this study; although, for two of them, this indication was very subtle. These indications varied among these participants but included high or relatively high pain and anxiety results in the adapted VAS-A questionnaire and negative feedback in the post-procedure interviews. In summary, the HRV results strongly indicate a positive effect from the VR intervention, namely, reduced stress levels and increased levels of relaxation, but also show some signs of a mixed effect for a 22% minority of the participants.

### 4.3. Interviews

The unstructured post-procedure interviews of the participants indicated a positive experience for 16 participants. Two participants provided neutral comments and one expressed a negative experience due to discomfort from the heart rate sensor and a disinterest in VR technology.

*“I felt that the exercise helped with the nervousness during the procedure. The exercise was easy to do, and I understood everything.”*

*(Participant, 10 years old)*

Regarding the effect of the VR intervention on cannulation, six participants shared their comments. Among them, five were positive, with four mentioning they did not notice or feel the needle puncture, and one expressing that the cannulation did not bother them. One participant noted that, while the VR experience was enjoyable, it did not help in the cannulation process. No participants reported symptoms of cybersickness [24].

*“I did not feel the puncture at all. It was fun.”*

*(Participant, 10 years old)*

Furthermore, a post-research interview with the head of the team of nurses suggested that the effectiveness of the VR intervention was strongly correlated to how well the patient was able to focus on the exercise.

### 4.4. Data Grouping Analysis

To gain further insights into the possible affective factors, the participants were divided into subgroups according to the following data: number of prior cannulations, VR experience, breathing exercise experience, level of needle phobia, and gender. These groups were compared regarding the data from the adapted VAS-A (expected/experienced pain/anxiety, pain/anxiety change) and HRV measurements (SDNN/RMSSD/SI/LFHF change). In addition, the interview results regarding general user experience were included to support the analysis, but were excluded from the statistical analysis.

Mann–Whitney U tests found no statistically significant differences in HRV variables between any of the paired subgroups, with the results being similarly positive for all of them. This is a significant result as it suggests a relatively equal stress-reducing effect, regardless of the participant background information variables studied in this data grouping analysis. Even so, the HRV variables were therefore excluded from the tables presented below.

Group divisions by prior cannulations are presented in Table 4. Mann–Whitney U tests were conducted to determine whether there were differences between the two groups divided by the level of prior cannulation experience. The results of these analyses indicate that the variables of expected pain (*z* = −3.204, *p* < 0.001), expected anxiety (*z* = −2.816, *p* = 0.006), and experienced anxiety (*z* = −2.668, *p* = 0.010) are greater for those with less prior experience regarding the procedure, and also present greater positive shifts between the variables of expected and experienced pain (*z* = 2.464, *p* = 0.013).

No statistically significant difference was achieved from the other analyzed variables. In other words, the data grouping analysis suggests that patients with little to no experience of the procedure tend to expect a more negative experience and also experience greater anxiety during the procedure. However, these patients also reported a greater decrease from expected to experienced pain, with experiences of pain being at the same level among the patients regardless of the number of previous experiences of the procedure.

Group division by previous VR experience is presented in Table 5. Mann–Whitney U tests found no statistically significant differences from the analyzed variables. However, it should be noted that the mean value of experienced pain was less than expected pain with no prior VR experience, while among those with previous VR experiences, the mean value of experienced pain was equal to expected pain.

Group division by previous experience from breathing exercises can be seen in Table 6. Mann–Whitney U tests found no statistically significant differences between the groups from any of the analyzed variables. However, it was noteworthy that all participants with prior breathing exercise experience expressed a positive experience of this study, whereas the results were more diverse among the participants without prior experience. In addition, the results from the adapted VAS-A questionnaire seem to be slightly more positive in the group with prior experience, although the difference is not statistically significant.

Group division by the estimated level of needle phobia can be found in Table 7. Kruskal–Wallis H tests indicated a statistically significant difference in expected pain, χ^2^(2) = 6.377, *p* = 0.041, pain change, χ^2^(2) = 6.774, *p* = 0.034, and anxiety change, χ^2^(2) = 8.274, *p* = 0.016, between the groups. Furthermore, differences in expected anxiety were close to statistical significance, χ^2^(2) = 5.577, *p* = 0.062.

Pairwise comparisons revealed a statistically significant difference in expected pain (*z* = −2.525, *p* = 0.035) between estimated needle phobia levels 0 and 2_,_ where pain change (*z* = 2.327, *p* = 0.060) and expected anxiety (*z* = −2.297, *p* = 0.065) were also relatively close to a statistically significant difference. Interestingly, the pairwise comparisons also revealed a statistically significant difference in anxiety change (*z* = 2.797, *p* = 0.015) between needle phobia levels 1 and 2, while no significant difference was observed between the other two pairs. Furthermore, all participants with clear indications of a needle phobia expressed a positive experience. In other words, patients with a needle phobia evaluated this method to be effective for addressing their treatment anxiety, whereas the subjective results were more mixed among patients with less need for such methods.

Finally, when grouped by gender, out of the 12 female participants, 10 expressed a positive experience while two expressed a neutral experience. Out of the seven male participants, six expressed a positive experience and one expressed a negative experience. Mann–Whitney U tests found no statistically significant differences between the genders from any of the analyzed variables.

### 4.5. Correlations

The SCARED average data were compared to other research data using a two-tailed Spearman’s rank-order correlation. Regarding the adapted VAS-A results, there was a statistically significant, moderate positive correlation with expected anxiety (*r_s_*(17) = 0.565, *p* = 0.012), but no statistically significant correlations were observed for the other variables. In other words, participants with high anxiety levels seemed to have also had high anxiety expectations regarding the procedure, but the anxiety levels of participants had no statistically significant correlation regarding the effect measured with the adapted VAS-A questionnaire.

In addition, the estimated level of needle phobia was compared to other research data using a two-tailed Spearman’s rank-order correlation. A statistically significant, moderate positive correlation was observed for expected pain (*r_s_*(17) = 0.595, *p* = 0.007) and expected anxiety (*r_s_*(17) = 0.532, *p* = 0.019), but no statistical significance was observed for experienced pain or anxiety. Furthermore, regarding the differences between the expectations and experiences, a statistically significant, moderate negative correlation was observed for pain change (*r_s_*(17) = −0.565, *p* = 0.012), and anxiety change (*r_s_*(17) = −0.445, *p* = 0.056) was close to a statistically significant, low negative correlation. This indicated that the level of needle phobia correlated with negative expectations regarding the procedure, but also with the positive effect measured with the adapted VAS-A questionnaire, especially regarding pain reduction.

Regarding the changes in the four key HRV measures, no statistically significant correlation was observed for the level of needle phobia. This implied that, according to the HRV data, the level of needle phobia did not affect the effect of the VR intervention.

## 5. Discussion

### 5.1. Main Results

The key finding of this study was the positive effect of deep breathing exercises in a virtual natural environment on ANS across the study population. The HRV results clearly show increased parasympathetic and decreased sympathetic nervous system activities during the VR intervention, which translates to reduced stress and anxiety levels and increased levels of relaxation. This effect was most likely caused by the conscious deep breathing performed by the child patients [7,34], the relaxing effect of experiencing a peaceful virtual natural environment [27,28,29,30], and the immersive, and thus distracting, nature of VR methods involving an HMD [15]. We considered this as a very significant finding, which was supported by participant interviews and positive results regarding user experiences in another article by the authors [9].

However, a 25% minority of the participants, including the two outliers, reported a negative experience in an interview or mixed effect in HRV variables, indicating that this method was not equally suitable for all patients. Furthermore, the adapted VAS-A answers indicated no significant difference between expected and experienced stress during the procedure regarding the whole study population. This could partially be due to the low level of stress measured for expectations, as the cannulation procedure was likely to produce some stress, but perhaps not too much, which left only little room for improvement.

It should be noted that, while the HRV measurements in this article studied only the effect of the VR intervention before the medical procedure, the adapted VAS-A results referred to the whole procedure, including the cannulation. Therefore, these results are not directly comparable. This contributes to the need for s further analysis of the HRV data regarding the medical procedure when considering the applicability of this method to address treatment anxiety in invasive pediatric procedures.

We found no evidence of a correlation between the positive effects measured in HRV variables to the background information, such as past experiences with VR devices or deep breathing exercises, the number of previous cannulations, the levels of anxiety or needle phobia of the participant, or gender. In other words, it seems like this method is effective regardless of these factors. However, according to the observations during the research, the positive effect is likely to be related to the ability or willingness to focus on the VR intervention.

In addition, the adapted VAS-A data revealed a stronger positive effect among participants who had higher levels of anxiety or needle phobia. Furthermore, the initial treatment anxiety (expectations) was higher among the participants with less experience regarding the procedure, who also experienced less pain than they expected. In other words, patients with an increased need for addressing treatment anxiety subjectively evaluated a greater positive impact. Also, previous deep breathing experiences contributed to a more positive effect, suggesting the potential for repeated use. Finally, no indications of simulation sickness [24] or other adverse effects were observed in the study.

### 5.2. Previous Studies

We were unable to find previous studies regarding the pediatric use of HMDs for deep breathing exercises in virtual natural environments. However, several studies have achieved positive effects when using HMD-based VR methods in pediatrics to address treatment anxiety. These studies focused on distraction by VR, and compared interactive VR application designs to more passive VR experiences, with the conclusions suggesting the inferiority of passive application designs for this purpose [3,4,55]. While our findings align with the positive effects of VR methods on pediatrics in general, the relatively passive approach of deep breathing in a virtual natural environment seems to be effective as well, suggesting that a focus on the level of interaction with the application can be misleading for pediatric purposes. A discussion regarding the design approaches for stress-reducing VR applications for pediatrics is presented in detail in another article by the authors [9].

Another similar study by Gerçeker et al. [6] compared two different VR contents to a control group during a blood draw, with one VR group experiencing a rollercoaster ride with the primary aim of distracting the patient, and the other VR group using more relaxing content, namely, an underwater tour with marine animals and soft music. Both approaches resulted in reduced pain and anxiety levels in comparison to the control group. Interestingly, there was no significant difference in the effect size regarding pain, but the relaxing VR content achieved a greater decrease in anxiety than the VR rollercoaster. This supports the idea of relaxing VR content being effective.

Some studies have found no significant stress-reducing effect from VR interventions in pediatrics. Custódio et al. [56] studied the use of cartoons with HMD for distraction during dental procedures, and while they observed good user acceptance with no adverse effects, there was no significant difference between the control group and the VR intervention group regarding patient behavior or reported pain. Similarly, Felemban et al. [57] used cartoons with HMD during anesthesia administration to reduce pain and anxiety levels, but observed no difference in the assessed pain between the VR intervention group and the control group. Eijlers et al. [58] used VR as a child-friendly preparation tool for elective day surgery, with VR content educating the patient about the upcoming procedures. Their results presented no significant differences regarding pain or anxiety between the VR intervention and control groups. It should be noted that none of these three studies were based on VR content directly aimed at stress reduction.

Recently, Prabhu et al. [59] studied a very similar VR method with 30 older adults for surgical anxiety and pain management in pre-operative and post-operative settings related to total knee arthroplasty. Their study also used a deep breathing exercise in a virtual natural environment and HRV data measurements, but it included a more advanced VR application design that incorporated biofeedback and gamification elements for guidance and motivation. This randomized control trial used a between-subjects design, with the participants assigned to control, 2D video (laptop computer screen), or VR (HMD) groups. Both 2D video and VR groups showed decreased pain and anxiety levels and increased parasympathetic activity in comparison to the control group, with the VR group exhibiting the greatest decrease in pain. This offers further support for the idea of using HMDs for deep breathing exercises in virtual natural environments in pediatrics.

The effect of the deep breathing exercises in a virtual natural environment is practically instantaneous according to the HRV data affecting the patient directly during the VR intervention. In contrast, a study using an interactive 360-degree art application presented increased stress levels during the experience, but also similar stress-reducing effects after the experience [41]. This highlights the need to identify the proper use of different VR relaxation methods in pediatrics. An immediate effect of the VR intervention is desirable when addressing treatment anxiety related to needle phobia, but some other medical procedures might benefit from a delayed effect, such as in the interactive art experience.

### 5.3. Implications and Recommendations

Utilizing guided deep breathing exercises in virtual natural environments proves to be an effective and pleasant non-pharmaceutical method for managing treatment anxiety in pediatrics. This approach holds great promise for improving patient satisfaction, particularly in invasive procedures, like intravenous cannulation. Further research using larger study samples, specifically targeting child patients with treatment anxiety, is highly recommended.

The stress-reducing effect of this method is almost immediate, which makes it potentially useful for any pediatric situation that would benefit from instant stress reduction. However, appropriate studies regarding the exact treatment scenario are recommended before applying these findings to other pediatric use cases.

While the method we studied was objectively effective, our results also identified two patient groups who benefitted less from it. The first group concerns patients who do not need anxiety reduction methods, and the second concerns patients who are unwilling or unable to focus on VR content. Therefore, this method should be considered as a tool to address treatment anxiety, rather than a standard method to be used for all child patients. All use cases should be voluntary.

### 5.4. Limitations

The study included a diverse, but relatively small, population, exhibiting lower anxiety levels compared to the general population. Since the studied method aims to decrease anxiety levels, additional research with a larger and more varied study population is necessary to assess the applicability of this approach in pediatrics, particularly for individuals with elevated anxiety levels.

Also, no control or comparison groups were used in this study. Therefore, it was impossible to separate the effect of the deep breathing exercise from the effect of experiencing a virtual natural environment, or from the possible excitement of experiencing novel VR technology. Furthermore, this article included no comparison to normal treatment without a VR intervention.

Patient diagnosis and the treatment path associated with intravenous cannulation were not included in the scope of this article. It is important to note that certain medical conditions may impact the effectiveness of this method.

In addition, this study provided no information beyond a speculation of how the repeated use of the method could affect the results, and the intervention protocol used in this research has not been published on any international intervention protocol platform.

Finally, the HRV data analysis in this article did not address the moment of cannulation or post-procedure phase of the research, and therefore could not be used as evidence of reduced pain and anxiety levels during that timeframe—it only contributed to showing the stress-reducing effect of deep breathing exercises in a virtual natural environment in this context.

## 6. Conclusions

The use of deep breathing exercises in a virtual natural environment shows strong potential for addressing treatment anxiety related to invasive pediatrics, with the results demonstrating a clear stress-reducing effect, regardless of the level of general anxiety or the level of needle phobia of the patient with no adverse effects. This potential is further supported by the good user-experience results among both the child patients and pediatricians.

This well-accepted method can be used to reduce a child’s stress related to his/her treatment. In turn, this can result in significantly improved treatment acceptance and patient satisfaction among patients with high stress levels.

However, further research on child patients suffering from treatment anxiety is highly recommended, as our study population was diverse, but relatively small, and the participants had relatively low mean levels of anxiety and needle phobia.

## Figures and Tables

**Table 1:** VirNE deep breathing exercise structure.

Timeframe	Exercise Phase Description	Dialog	Breathing Animation	Marked for Procedure
0:00–0:30	The selected virtual natural environment starts to play, and the selected avatar character is present	-	-	-
0:30–1:00	The exercise starts. Introduction to the exercise and setting of body posture	**X**	-	-
1:00–2:00	Teaching conscious, slow breathing. Breathing animation appears	**X**	**X**	-
2:00–	Guided, slow, deep breathing for 30 breathing cycles of 6 s starts. Short, encouraging dialog cues are played periodically, once per 30 s	**X**	**X**	-
3:30–4:00	The period marked for conducting the cannulation, visible only to the nurse controlling the application with the PC	-	**X**	**X**
–5:00	Guided, slow, deep breathing continues until 5:00	**X**	**X**	-
5:00–5:30	Returning to normal breathing and ending the exercise. The breathing animation disappears	**X**	-	-
5:30–6:00	The virtual environment continues to play for 30 more seconds. This period aims for a smooth ending of the experience, and the removal of the head-mounted display is conducted during it	-	-	-

**Table 2:** Demographics of the child patients included in the statistical results.

Data Group	Study Sample
N	19 participants
Age (mean [SD])	10.1 (±1.29) years old
Gender division	12 females, 7 males
Height (mean [SD])/weight (mean [SD])	144.0 (±11.8) cm/38.2 (±10.5) kg
Prior intravenous cannulations	
*0–2 cannulations*	*10 participants*
*3+ cannulations*	*9 participants*
Level of needle phobia	
*0—no needle phobia*	*7 participants*
*1—minor needle phobia*	*6 participants*
Prior virtual reality experience	11 had experience, 8 did not
Prior deep breathing experience	11 had experience, 8 did not
Sensitivity to motion sickness	
*Low/medium/high*	*9/6/4 (participants)*

**Table 3:** This table presents the median values and interquartile ranges of HRV variables in two conditions. All four HRV variables indicate a stress-reducing effect when comparing the VR intervention to baseline measurement.

HRV Variable (Unit)	Sample 1: Baseline Mdn (IQR)	Sample 2: VR Intervention Mdn (IQR)
HR (bpm)	87.50 (16.50)	85.00 (18.75)
SDNN (ms)	35.65 (19.77)	62.70 (26.13)
RMSSD (ms)	32.15 (29.88)	53.50 (40.18)
Stress Index	14.00 (6.18)	8.40 (4.02)
LF/HF ratio	1.554 (2.080)	0.456 (0.550)

**Table 4:** Group division by prior cannulations.

Data Group	0–2 Prior Cannulations	3+ Prior Cannulations
N	10	9
Interview (UX): pos./neut./neg.	9/0/1	7/2/0
Pain: expectation mean (SD)	3.40 (±1.51)	1.11 (±0.60)
Pain: experience mean (SD)	2.20 (±1.69)	1.67 (±0.87)
Pain: change mean (SD)	−1.20 (±1.69)	0.56 (±0.78)
Anxiety: expectation mean (SD)	2.10 (±1.37)	0.56 (±0.73)
Anxiety: experience mean (SD)	2.10 (±1.74)	0.44 (±1.01)
Anxiety: change mean (SD)	0.00 (±1.63)	−0.11 (±0.78)

**Table 5:** Group division by prior VR experience.

Data Group	VR Experience: No	VR Experience: Yes
N	8	11
Interview (UX): pos./neut./neg.	7/0/1	9/2/0
Pain: expectation mean (SD)	2.50 (±1.77)	2.18 (±1.60)
Pain: experience mean (SD)	1.63 (±1.60)	2.18 (±1.17)
Pain: change mean (SD)	−0.88 (±1.81)	0.00 (±1.48)
Anxiety: expectation mean (SD)	1.50 (±1.31)	1.27 (±1.42)
Anxiety: experience mean (SD)	1.75 (±2.12)	1.00 (±1.18)
Anxiety: change mean (SD)	0.25 (±1.67)	−0.27 (±0.90)

**Table 6:** Group division by prior deep breathing experience.

Data Group	Deep Breathing Experience: No	Deep Breathing Experience: Yes
N	8	11
Interview (UX): pos./neut./neg.	5/2/1	11/0/0
Pain: expectation mean (SD)	2.13 (±1.73)	2.45 (±1.63)
Pain: experience mean (SD)	2.13 (±1.46)	1.82 (±1.33)
Pain: change mean (SD)	0.00 (±1.31)	−0.64 (±1.86)
Anxiety: expectation mean (SD)	1.50 (±1.31)	1.27 (±1.42)
Anxiety: experience mean (SD)	2.00 (±2.14)	0.82 (±0.98)
Anxiety: change mean (SD)	0.50 (±1.60)	−0.45 (±0.82)

**Table 7:** Group division by the estimated level of needle phobia.

Data Group	0 = No Needle Phobia	1 = Minor Needle Phobia	2 = Clear Needle Phobia
N	7	6	6
Interview (UX): pos./neut./neg.	6/1/0	4/1/1	6/0/0
Pain: expectation mean (SD)	1.29 (±0.95)	2.33 (±1.86)	3.50 (±1.38)
Pain: experience mean (SD)	2.14 (±1.21)	1.50 (±1.64)	2.17 (±1.33)
Pain: change mean (SD)	0.86 (±1.07)	−0.83 (±1.17)	−1.33 (±1.86)
Anxiety: expectation mean (SD)	0.71 (±0.76)	1.00 (±0.89)	2.50 (±1.64)
Anxiety: experience mean (SD)	0.71 (±1.11)	1.83 (±2.32)	1.50 (±1.38)
Anxiety: change mean (SD)	0.00 (±0.82)	0.83 (±1.60)	−1.00 (±0.63)

## Data Availability

A summary of the data presented in this study is presented within the article. Full dataset not available due to ethical and privacy issues.
